# Supplementary material for: Computationally guided in-vitro vascular growth model reveals causal link between flow oscillations and disorganized neotissue
Source: Commun Biol. 2021 May 10;4:546. doi: 10.1038/s42003-021-02065-6 (PMC8110791; doi:10.1038/s42003-021-02065-6)
Supplement: Supplementary file 2 — Supplementary Information [file 42003_2021_2065_MOESM2_ESM.pdf]

SUPPLEMENTARY INFORMATION
TO
COMPUTATIONALLY GUIDED IN-VITRO VASCULAR GROWTH MODEL REVEALS CAUSAL LINK BETWEEN FLOW OSCILLATIONS AND DISORGANIZED NEOTISSUE

ELINE E. VAN HAAFTEN[1,2], SJENG QUICKEN[3], WOUTER HUBERTS[3], CARLIJN V.C. BOUTEN[1,2] ✉, AND NICHOLAS A. KURNIAWAN[1,2]

✉ C.V.C.Bouten@tue.nl

[1] Department of Biomedical Engineering, Eindhoven University of Technology, Eindhoven, The Netherlands

[2] Institute for Complex Molecular Systems (ICMS), Eindhoven University of Technology, Eindhoven, The Netherlands

[3] Department of Biomedical Engineering, CARIM School for Cardiovascular Diseases, Maastricht University, Maastricht, The Netherlands

Date: March 24, 2021.

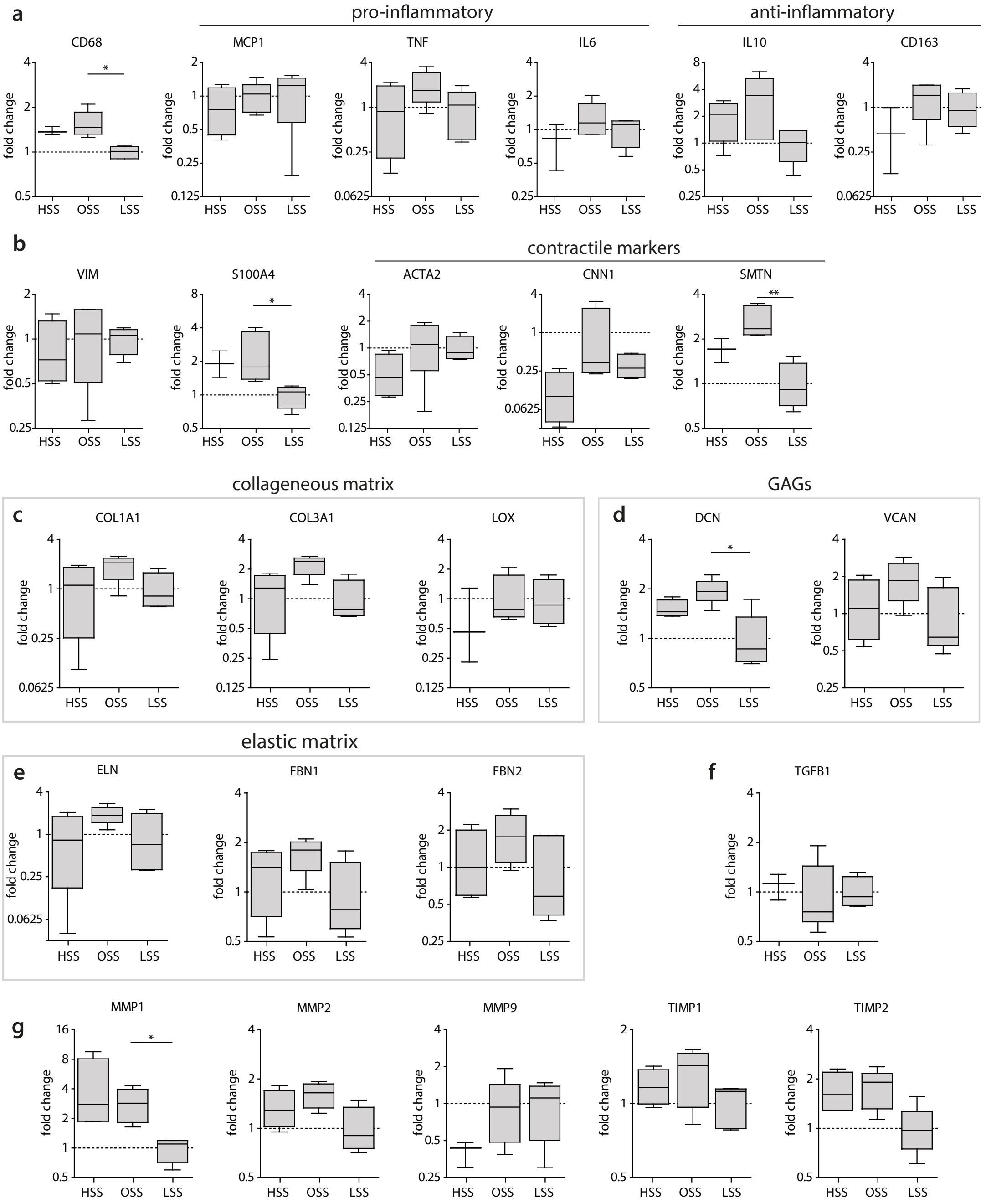

Supplementary Figure S1. **Gene expression analysis of phenotypical, growth, and remodeling markers after 14 days of dynamic culture.** Relative gene expression compared to the low shear stress condition for **(a)** macrophage-related genes, **(b)** (myo)fibroblast-related genes, **(c)** collageneous matrix- related genes, **(d)** GAGs-related genes, **(e)** elastic matrix-related genes, **(f)** TGFB1, and **(g)** proteases. HSS, high shear stress condition (3.2 Pa); OSS, oscillatory shear stress condition (±3.2 Pa); LSS, low shear stress condition (0.4 Pa) ($n = 3$, 4, or 5/group, * $p < 0.05$, ** $p < 0.01$).

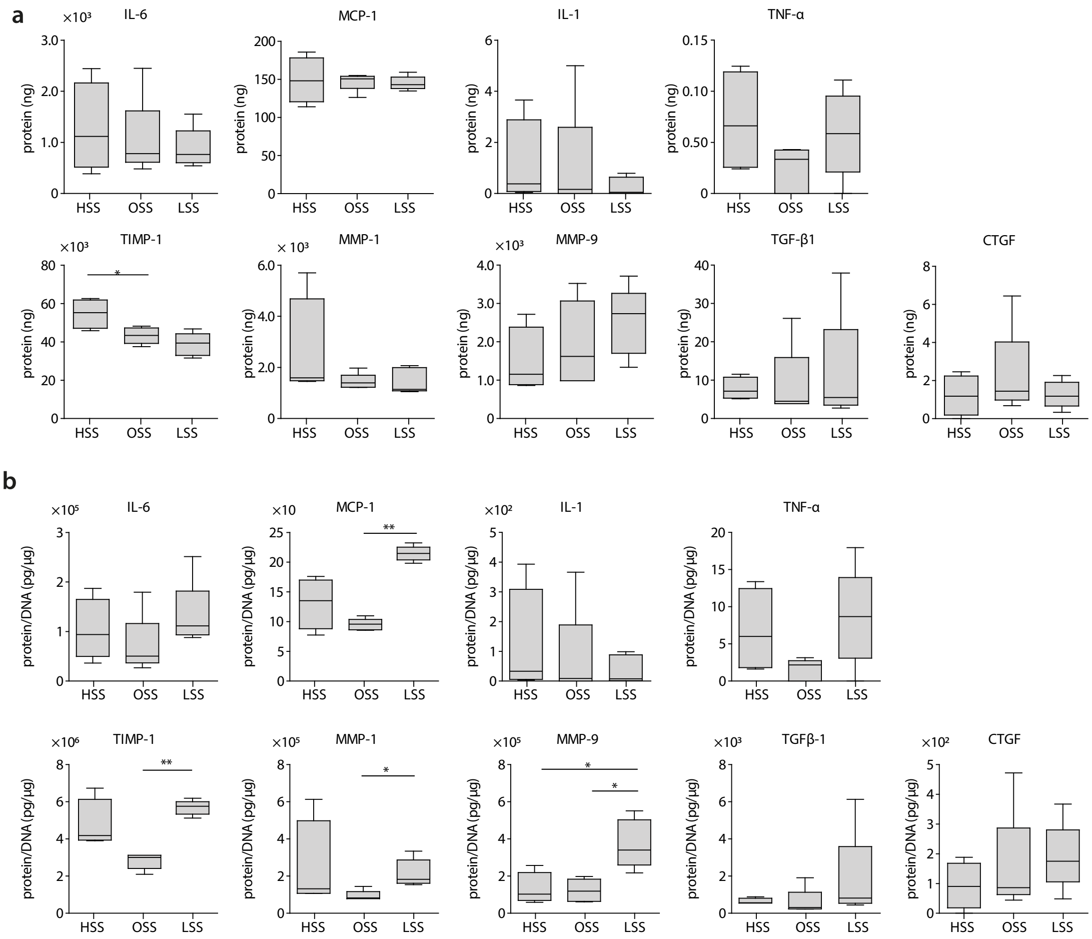

SUPPLEMENTARY FIGURE S2. **Secretion profiles of intimal hyperplasia- and tissue formation-related proteins at day 14. (a)** Total protein production in the culture medium and **(b)** protein production in the culture medium normalized to DNA content. HSS, high shear stress condition (3.2 Pa, $n = 4$); OSS, oscillatory shear stress condition (±3.2 Pa, $n = 5$); LSS, low shear stress condition (0.4 Pa, $n = 5$) (* $p < 0.05$, ** $p < 0.01$).

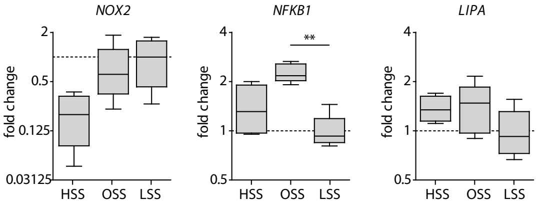

Supplementary Figure S3. **Gene expression analysis of degradation after 14 days of dynamic culture.** HSS, high shear stress condition (3.2 Pa); OSS, oscillatory shear stress condition (±3.2 Pa); LSS, low shear stress condition (0.4 Pa) (n = 4 or 5/group, * $p < 0.05$, ** $p < 0.01$).

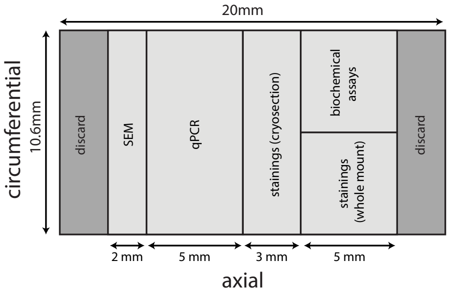

Supplementary Figure S4. **Cutting scheme for day 14 scaffolds.**

Primer	*Symbol*	*Accession number*	*Primer sequence ('5-'3)*
Phenotypic markers			
Cluster of differentiation 68	*CD68*	NM_001040059.1	FW: CTACTGGCAGAGAGCACTGG<br>RV: CCGCCATGTAGCTCAGGTAG
Cluster of differentiation 163	*CD163*	NM_004244	FW:CACTATGAAGAAGCCAAAATTACCT<br>RV: AGAGAGAAGTCCGAATCACAGA
Monocyte chemoattractant protein 1	*MCP1*	NM_002982	FW: CAGCCAGATGCAATCAATGCC<br>RV: TGGAATCCTGAACCCACTTCT
Tumor necrosis factor α	*TNF*	NM_000594	FW: GAGGCCAAGCCCTGGTATG<br>RV: CGGGCCGATTGATCTCAGC
Interleukin 6	*IL6*	NM_000600	FW: ACTCACCTCTTCAGAACGAATTG<br>RV: GTCGAGGATGTACCGAATTTGT
Interleukin 10	*IL10*	NM_000572	FW: GACTTTAAGGGTTACCTGGGTTG<br>RV: TCACATGCGCCTTGATGTCTG
α smooth muscle actin	*ACTA2*	NM_001613.1	FW: CGTGTTGCCCCTGAAGAGCAT<br>RV: ACCGCCTGGATAGCCACATACA
Smoothelin	*SMTN*	NM_134270	FW: CAGCCCAGAACCGAGAGTC<br>RV: AGCAGCCATAGGAGAATCAGAT
Calponin	*CNN1*	NM_001299.5	FW:TGAAGTACGCAGAGAAGCAG<br>RV:CAGCTTGGGGTCGTAGAG
S100 calcium binding protein A4	*S100A4*	NM_002961	FW:TCTTTCTTGGTTTGATCCTGACT<br>RV: AGTTCTGACTTGTTGAGCTTGA
Vimentin	*VIM*	NM_003380	FW: AAGACCTGCTCAATGTTAAGATC<br>RV: CTGCTCTCCTCGCCTTCC
Tissue formation			
Transforming growth factor β	*TGFB1*	NM_000660	FW: GCAACAATTCCTGGCGATACCTC<br>RV: AGTTCTTCTCCGTGGAGCTGAAG
Collagen type I	*COL1A1*	NM_000088	FW: AATCACCTGCGTACAGAACGG<br>RV: TCGTCACAGATCACGTCATCG
Collagen type III	*COL3A1*	NM_000090	FW: ATCTTGGTCAGTCCTATGC<br>RV: TGGAATTTCTGGGTTGGG
Lysyl oxidase	*LOX*	NM_002317.3	FW:CCTGGCTGTTATGATAC<br>RV: GAGGCATACGCATGATG
Elastin	*ELN*	NM_000501.3	FW: CTGGAATTGGAGGCATCG<br>RV: TCCTGGGACACCAACTAC
Fibrillin 1	*FBN1*	NM_00138	FW:TGTTGGTTTGTGAAGATATTG<br>RV: GTGGAGGTGAAGCGGTAG
Fibrillin 2	*FBN2*	NM_001999	FW:ATCCCTGTGAGATGTGTC<br>RV: TTCCTCCTTGGCATATCC
Decorin	*DCN*	NM_133503	FW:TGCAGCTAGCCTGAAAGGAC<br>RV: TTGGCCAGAGAGCCATTGTC
Versican	*VCAN*	NM_004385	FW:GGCACCTGTTATCCTACTGAAA<br>RV: ACACAAGTGGCTCCATTACG
Tissue remodeling			
Matrix metalloproteinase 1	*MMP1*	NM_001145938.1	FW:CGCACAAATCCCTTCTACCC<br>RV: CTGTCGGCAAATTCGTAAGC
Matrix metalloproteinase 2	*MMP2*	NM_001127891	FW: ATGACAGCTGCACCACTGAG<br>RV: ATTTGTTGCCCAGGAAAGTG
Matrix metalloproteinase 9	*MMP9*	NM_004994	FW: TGGGGGGCAACTCGGC<br>RV: GGAATGATCTAAGCCCAG
Metallopeptidase inhibitor 1	*TIMP1*	NM_003254.2	FW: TGACATCCGGTTCGTCTACA<br>RV: TGCAGTTTTCCAGCAATGAG
Metallopeptidase inhibitor 2	*TIMP2*	NM_003255.4	FW:GGAGGAATCGGTGAGGTC<br>RV: AACAGGCAAGAACAATGG
Construct degradation			
Nicotinamide adenine dinucleotide phosphate-oxidase 2	*NOX2*	NM_000397.3	FW:AACTGGGCTGTGAATGAGGG<br>RV: GCCAGTGCTGACCCAAGAA
Nuclear factor κ-light-chain-enhancers of activated B cells	*NFKB1*	NM_001165412	FW:AGACCAAGGAGATGGACCTCA<br>RV: GCATTGGGGGCTTTACTGTC
Lipase A or cholesterol ester hydrolase	*LIPA*	NM_001288979.1	FW:TCCTGCTGGAACTTCTGTGC<br>RV: ACTGCTTCCCCAGTCAAAGG

Supplementary Table S1. Primers used for gene expression analysis

Protein	*Symbol*	*Function*	*qPCR*	*ELISA*
Phenotypic markers				
Cluster of differentiation 68	CD68	Pan-macrophage marker	×	
Cluster of differentiation 163	CD163	Anti-inflammatory macrophage marker	×	
Monocyte chemoattractant protein 1	MCP-1	Chemotactic for monocytes/macrophages	×	×
Tumor necrosis factor α	TNF-α	Pro-inflammatory factor, stimulus for collagen production, inhibitor of elastogenesis	×	×
Interleukin 1	IL-1	Neutrophil/monocyte recruitment		×
Interleukin 6	IL-6	Pro-inflammatory factor	×	×
Interleukin 10	IL-10	Anti-inflammatory cytokine, inhibitor of collagen production	×	
α smooth muscle actin	αSMA	Filament of the cytoskeleton involved in regulating cell shape, movement and involved in cell contractility	×	
Smoothelin	SMTN	Constitutes part of the cytoskeleton and is found exclusively in contractile smooth muscle cells	×	
Calponin	CNN1	Protein that is involved in the modulation and regulation of smooth muscle cell contraction	×	
S100 calcium binding protein A4	S100A4	Protein involved in the regulation of multiple cellular processes (e.g., cell cycle progression, differentiation, tubulin polymerization). Activated fibroblast, regulated to tissue remodeling	×	
Vimentin	VIM	Intermediate filament protein, part of the cytoskeleton, used as fibroblast marker	×	
Tissue formation				
Transforming growth factor β	TGF-β1	Anti-inflammatory factor; stimulus for collagen formation	×	×
Connective tissue growth factor	CTGF	Stimulus for collagen formation		×
Collagen type I	COL1A1	Load bearing protein of the extracellular matrix	×	
Collagen type III	COL3A1	A fibrillary collagen; found frequently in association with type I collagen	×	
Lysyl oxidase	LOX	Enzyme involved in collagen and elastin crosslinking	×	
Elastin	ELN	Tropoelastin, one of the main components of the elastic fiber	×	
Fibrillin 1	FBN-1	Extracellular matrix protein that provides structural support for elastic fibril formation	×	
Fibrillin 2	FBN-2	Extracellular matrix protein that provides structural support for elastic fibril formation	×	
Decorin	DCN	Proteoglycan important in collagen fibril assembly	×	
Versican	VCAN	Proteoglycan important for cell adhesion, proliferation, differentiation and migration. Proven important for elastic network formation	×	
Remodeling				
Matrix metalloproteinase 1	MMP-1	Extra-cellular breakdown and remodeling	×	×
Matrix metalloproteinase 2	MMP-2	Extra-cellular breakdown and remodeling	×	
Matrix metalloproteinase 9	MMP-9	Anti-inflammatory factor involved in extra-cellular breakdown and remodeling	×	×
Metallopeptidase inhibitor 1	TIMP-1	Inhibitor of MMPs	×	×
Metallopeptidase inhibitor 2	TIMP-2	Inhibitor of MMPs	×	
Degradation				
Nicotinamide adenine dinucleotide phosphate-oxidase 2	NOX2	Enzyme complex that contributes to ROS production	×	
Nuclear factor κ-light-chain-enhancers of activated B cells	NF$\kappa\beta$	Involved in cellular responses to oxidative stress and cell survival	×	
Lipase A or cholesterol ester hydrolase	LIPA	Lysosomal enzyme	×	

Supplementary Table S2. Genes and proteins analyzed via qPCR (primer sequences in Supplementary Table S1) and Multiplex ELISA
