# Supplementary material for: Computationally guided in-vitro vascular growth model reveals causal link between flow oscillations and disorganized neotissue
Source: Commun Biol. 2021 May 10;4:546. doi: 10.1038/s42003-021-02065-6 (PMC8110791; doi:10.1038/s42003-021-02065-6)
Supplement: Supplementary file 3 — Description of Additional Supplementary Files [file 42003_2021_2065_MOESM3_ESM.pdf]

Description of Additional Supplementary Files

File Name: Supplementary Data 1
Description: Supplementary Data containing all source data underlying the graphs and charts presented in "Computationally guided in-vitro vascular growth model reveals causal link between flow oscillations and disorganized neotissue"
