# Supplementary material for: Computationally guided in-vitro vascular growth model reveals causal link between flow oscillations and disorganized neotissue
Source: Commun Biol. 2021 May 10;4:546. doi: 10.1038/s42003-021-02065-6 (PMC8110791; doi:10.1038/s42003-021-02065-6)
Supplement: Supplementary file 5 — Reporting Summary [file 42003_2021_2065_MOESM5_ESM.pdf]

nature research

Corresponding author(s): Carlijn Bouten

Last updated by author(s): Feb 7, 2021

Reporting Summary

Nature Research wishes to improve the reproducibility of the work that we publish. This form provides structure for consistency and transparency in reporting. For further information on Nature Research policies, see our Editorial Policies and the Editorial Policy Checklist.

Statistics

For all statistical analyses, confirm that the following items are present in the figure legend, table legend, main text, or Methods section.

n/a	Confirmed	
☐	☒	The exact sample size (*n*) for each experimental group/condition, given as a discrete number and unit of measurement
☐	☒	A statement on whether measurements were taken from distinct samples or whether the same sample was measured repeatedly
☐	☒	The statistical test(s) used AND whether they are one- or two-sided<br>*Only common tests should be described solely by name; describe more complex techniques in the Methods section.*
☒	☐	A description of all covariates tested
☐	☒	A description of any assumptions or corrections, such as tests of normality and adjustment for multiple comparisons
☐	☒	A full description of the statistical parameters including central tendency (e.g. means) or other basic estimates (e.g. regression coefficient) AND variation (e.g. standard deviation) or associated estimates of uncertainty (e.g. confidence intervals)
☐	☒	For null hypothesis testing, the test statistic (e.g. *F*, *t*, *r*) with confidence intervals, effect sizes, degrees of freedom and *P* value noted<br>*Give P values as exact values whenever suitable.*
☒	☐	For Bayesian analysis, information on the choice of priors and Markov chain Monte Carlo settings
☒	☐	For hierarchical and complex designs, identification of the appropriate level for tests and full reporting of outcomes
☒	☐	Estimates of effect sizes (e.g. Cohen's *d*, Pearson's *r*), indicating how they were calculated

Our web collection on statistics for biologists contains articles on many of the points above.

Software and code

Policy information about availability of computer code

Data collection	Computational part: OASIS (CFD solver), UNICORN (FSI solver), FEniCS (finite element package), Python (to run the CFD simulations), c++ (to run the FSI simulations), Matlab (postprocessing); experimental part: equipment-specific software (i.e., ARES rheometer, Qubit DNA assay, microplate reader, Quanta 600F SEM, Leica TCS SP5X, Zeiss Axiovert microscope, Zeiss Axio Observer Z1, NanoDrop spectrophotometer, Bio-Rad Thermal Cycler, Bio-Plex Manager Software), Matlab (postprocessing collagen z-stacks)
Data analysis	Computational part: ParaView (result visualization in Fig. 1), Matlab (result visualization in Fig. 1); experimental part: Prism (statistics, bar- and boxplots), Matlab (boxplots in Fig. 2 and all other graphs)

For manuscripts utilizing custom algorithms or software that are central to the research but not yet described in published literature, software must be made available to editors and reviewers. We strongly encourage code deposition in a community repository (e.g. GitHub). See the Nature Research guidelines for submitting code & software for further information.

Data

Policy information about availability of data

All manuscripts must include a data availability statement. This statement should provide the following information, where applicable:

- Accession codes, unique identifiers, or web links for publicly available datasets
- A list of figures that have associated raw data
- A description of any restrictions on data availability

The data that support the findings of this study are available from the corresponding author upon reasonable request.

April 2020

Field-specific reporting

Please select the one below that is the best fit for your research. If you are not sure, read the appropriate sections before making your selection.

- [x] Life sciences
- [ ] Behavioural & social sciences
- [ ] Ecological, evolutionary & environmental sciences

For a reference copy of the document with all sections, see nature.com/documents/nr-reporting-summary-flat.pdf

Life sciences study design

All studies must disclose on these points even when the disclosure is negative.

Sample size	computational part: the model geometry was derived from 1 patient dataset, and simulations were performed with a single set of boundary conditions, as this model was considered representative to obtain the order of magnitude of the output parameters of interest;<br><br>experimental part: sample sizes were chosen to allow for statistical comparisons (i.e., at least n=3/condition but preferable n=5/condition):<br>- SEM (construct), N=5 per loading condition (N=4 for HSS), 3 technical replicates/sample, note: 1 overview picture and 3 zoomed-in pictures per sample<br>- qPCR (construct), N=5 per loading condition (N=4 for HSS), 2 technical replicates/sample<br>- Stainings (cryosections) (construct), N=5 per loading condition (N=4 for HSS), 3 technical replicates/sample, note: 3 sections per sample<br>- Stainings (whole mount) (construct), N=5 per loading condition (N=4 for HSS), 2 technical replicates/sample, note: 1 overview picture and 2 zoomed-in pictures per sample<br>- Biochemical assays (construct), N=5 per loading condition (N=4 for HSS), 2 technical replicates/sample<br>- ELISA (medium), N=5 per loading condition (N=4 for HSS), 1 technical replicate/sample<br>- Viscosity (medium), N=5 per time point, 1 technical replicate/sample, note: a selection of samples from different loading conditions were grouped together<br>- Fiber analysis (construct), N=1 per loading condition, 30 technical replicates/sample, note: analysis on SEM pictures of 1 selected sample for decellularization per loading condition<br>- Stretch (repeated measure during culture), N=13-14 per time point, 1 technical replicate/sample, note: all samples from different loading conditions were grouped together<br>- Flow (repeated measure during culture), N=9 (HSS and OSI) and N=6-7 (LSS) per time point, 1 technical replicate/sample, note: all samples from HSS and OSI condition were grouped together.
Data exclusions	1 sample from the HSS condition was discarded from all analyses because of bioreactor failure during the culture period. In the qPCR analysis, datapoints were excluded if: 1) melting curve off 2) duplicate >0.5 Ct difference 3) blanco off in comparison to measured Ct value
Replication	The culture experiment was repeated twice and no experimental-specific outcomes were observed
Randomization	After mounting the cell-seeded scaffolds in the bioreactor culture chambers, the samples were allocated to the different experimental conditions. This occurred in a non-blinded, random way.
Blinding	Investigators of the ELISA and qPCR analysis were blinded. In other quantitative analyses, investigators were not blinded. This was not considered relevant because of the objectivity of the data. In microscopical analyses, investigators were not blinded, but images were taken at multiple, pre-defined locations, to get a representative and objective view of each sample.

Reporting for specific materials, systems and methods

We require information from authors about some types of materials, experimental systems and methods used in many studies. Here, indicate whether each material, system or method listed is relevant to your study. If you are not sure if a list item applies to your research, read the appropriate section before selecting a response.

Materials & experimental systems

n/a	Involved in the study	
☐	☒	Antibodies
☐	☒	Eukaryotic cell lines
☒	☐	Palaeontology and archaeology
☒	☐	Animals and other organisms
☒	☐	Human research participants
☐	☒	Clinical data
☒	☐	Dual use research of concern

Methods

n/a	Involved in the study	
☒	☐	ChIP-seq
☒	☐	Flow cytometry
☒	☐	MRI-based neuroimaging

Antibodies

Antibodies used	Primary antibodies for fluorescence stainings:<br>Ki67, rabbit IgG, Thermoscientific, RB-1510-P0<br>CD45, mouse IgG1, Abcam, ab33533<br>vimentin, mouse IgM, Abcam, ab20346<br>aSMA, rabbit IgG, Abcam, ab5694<br>collagen I, mouse IgG, Sigma, c2456<br><br>Secondary antibodies for fluorescence stainings:<br>goat-antimouse IgG1-488, Molecular Probes, A21121<br>goat-antirabbit IgG-488, Molecular Probes, A11008<br>goat-antimouse IgM-647, Jackson Immunoresearch, 115-605-075<br>goat-antimouse IgG1-647, Molecular Probes, A21240
Validation	All antibodies have been validated as reported by the manufacturers and in our previous studies.

Eukaryotic cell lines

Policy information about cell lines

Cell line source(s)	THP1 Sigma Aldrich, lot# 16K052
Authentication	Authentication of the cell line was performed by the supplier prior to shipping, as reported in the Certificate of Analysis associated with the lot number.
Mycoplasma contamination	The cells tested negative on routinely performed (monthly) mycoplasm tests.
Commonly misidentified lines<br>(See ICLAC register)	No commonly misidentified lines were used in this study.

Clinical data

Policy information about clinical studies

All manuscripts should comply with the ICMJEguidelines for publication of clinical research and a completedCONSORT checklist must be included with all submissions.

Clinical trial registration	N/A
Study protocol	N/A
Data collection	N/A
Outcomes	N/A
